# Spontaneous bilateral femoral neck fractures in a young male adult: a case report and literature review

**DOI:** 10.1186/s12891-019-2857-9

**Published:** 2019-10-15

**Authors:** Shinkichi Arisumi, Taro Mawatari, Satoshi Ikemura, Gen Matsui, Takahiro Iguchi, Hiroaki Mitsuyasu

**Affiliations:** Department of Orthopaedic Surgery, Hamanomachi Hospital, 3-3-1 Nagahama, Chuo-ku, Fukuoka, 810-8539 Japan

**Keywords:** Spontaneous femoral neck fracture, Bilateral, Osteoporosis, Vitamin D insufficiency, MRI, Case report

## Abstract

**Background:**

Simultaneous bilateral femoral neck fracture is a very rare condition, even in osteoporotic elderly individuals. We report an atypical case of a young male adult who developed simultaneous bilateral femoral neck fractures without previous trauma or overuse.

**Case presentation:**

A 33-year-old man presented with discomfort in the bilateral groin, which had started 2 weeks previously. Bilateral femoral neck fractures were observed on a radiograph, and in addition, a fracture line was seen at the right subchondral region of the acetabulum using magnetic resonance imaging (MRI). Although the patient had no obvious risk factors associated with bone fragility, his bone mineral density measured using dual X-ray absorptiometry indicated severe osteoporosis (lumber spine: T score − 3.4 standard deviation [SD]; femoral neck: T score − 2.8 SD). Serum 25-hydroxyvitamin D level was deficient (19 ng/mL), which was considered to be partly due to non-sunlight exposure for 3 years owing to social withdrawal. Bilateral osteosynthesis was performed, considering his young age, although more than 2 weeks had passed since the onset of the fracture. Bone union and non-occurrence of osteonecrosis of the femoral head were confirmed via radiography and MRI 8 months after the surgery.

**Conclusions:**

Our case suggests that simultaneous non-traumatic bilateral femoral neck fractures can occur in healthy young men.

## Background

Femoral neck fractures in the elderly are among the most commonly experienced orthopedic trauma, but the incidence in the young population is rare. Hip fractures among young people are usually seen in instances of high-energy trauma, single episodes of high-level stress, or repetitive microinjury leading to stress fractures, such as those as commonly observed in the military population and in persons in metabolically altered bone states [1–4]. Non-traumatic bilateral femoral neck fractures are very rare. Only a few cases of bilateral femoral neck fractures secondary to transient osteoporosis of the hip in postpartum patients have also been described in the literature [5, 6].

We recently treated a young male adult who developed simultaneous bilateral femoral neck fractures without an antecedent trauma.

## Case presentation

A 33-year-old male patient started experiencing discomfort in the bilateral groin without any history of trauma and visited our outpatient clinic 2 weeks after the onset. He had begun a routine of jogging 2 km/week, a month previously. A medical interview revealed that he had become socially withdrawn and had not gone out during the day for 3 years, and he jogged after dusk only. Thus, he had not received sunlight exposure for 3 years. We considered that he had no obvious risk factors associated with bone fragility, such as type II diabetes, chronic kidney disease, rheumatoid arthritis, alcohol consumption, or corticosteroid, anticoagulant, or antipsychotic medication use [7], based on the results of laboratory tests or medical interview. His body mass index was 18.2 kg/m^2^ (normal range: 18.5–25.0 kg/m^2^) [8]. Bone mineral density measured using dual X-ray absorptiometry in the lumbar spine and in the left femoral neck were 0.639 g/cm^2^ (T score: − 3.4 standard deviation [SD]) and 0.512 g/cm^2^ (T score: − 2.8 SD), respectively, which were considered indicative of severe osteoporosis. The patient could walk, but with discomfort, and a non-antalgic gait was observed. The ranges of motion of both hips were 120° flexion, 0° extension, 30° abduction, 20° adduction, 30° external rotation, and 30° internal rotation, which were within the normal ranges.

The results of laboratory tests that were conducted during his first visit to our hospital are summarized in Table 1. An examination of the patient’s bone metabolism revealed decreased 25-hydroxyvitamin D [25(OH)D] level (19 ng/mL) [9–11] and an elevated P1NP level (84.8 ng/mL), but calcium and phosphorus levels were within the normal ranges. The patient’s rheumatoid factor and anti-cyclic citrullinated peptide antibody were also within the normal ranges. Regarding nutrition status, the total protein (7.2 g/dL) and albumin (4.4 g/dL) levels were also within the normal ranges. Although his serum squamous cell carcinoma level (3.0 ng/mL) was slightly elevated, no abnormal finding was observed in the chest computed tomography (CT) scan.

**Table 1:** Laboratory data at patient’s first visit

Parameter	Value		Normal range
Na	141 mEq/L		135–148 mEq/L
K	4.1 mEq/L		3.6–5.2 mEq/L
Cl	105 mEq/L		98–108 mEq/L
Ca	9.4 mg/dL		8.4–10.2 mg/dL
P	3.8 mg/dL		3.0–4.7 mg/dL
TP	7.2 g/dL		6.5–8.1 g/dL
Alb	4.4 g/dL		4.1–5.1 g/dL
ALP	414 U/L	(H)	100–330 U/L
Hb A1c	5.5%		4.9–6.0%
CEA	1.9 ng/ml		≤5.0 ng/ml
CA19–9	11 U/ml		≤37.0 U/ml
PSA	0.156 ng/ml		< 4.0 ng/ml
SCC	3.0 ng/ml	(H)	≤1.5 ng/ml
CRP	0.68 mg/dl	(H)	0.00–0.14 mg/dl
RF	< 3.0 IU/ml		< 15 IU/ml
ACPA	0.7 U/ml		< 4.4 U/ml
FT4	1.35 ng/mL		0.70–1.48 ng/mL
TSH	1.70 μIU/mL		0.34–4.94 μIU/mL
PTH	25 pg/mL		10–60 pg/mL
P1NP	84.8 μg/L	(H)	19.5–71.2 μg/L
OC	4.07 ng/mL	(L)	8.3–32.7 ng/mL
25(OH)D	19 ng/mL	(L)	
TRACP-5b	255 mU/dL		170–590 mU/dL

*Na* sodium, *K* potassium, *Cl* chlorine, *Ca* calcium, *P* phosphorus, *TP* total protein, *Alb* albumin, *ALP* alkaline phosphatase, *Hb A1c* hemoglobin A1c, *CEA* carcinoembryonic antigen, *CA19–9* carbohydrate antigen 19–9, *PSA* prostate specific antigen, *SCC* squamous cell carcinoma antigen, *CRP* C-reactive protein, *RF* rheumatoid factor, *ACPA* anti-cyclic citrullinated peptide antibody, *FT4* free thyroxine 4, *TSH* thyroid-stimulating hormone *PTH* parathyroid hormone, *P1NP* type I procollagen N-terminal propeptide, *OC* osteocalcin, *25(OH) D* hydroxyvitamin D, *TRACP-5b* tartrate-resistant acid phosphatase 5b, *(H)* high, *(L)* low

**Fig. 1:**
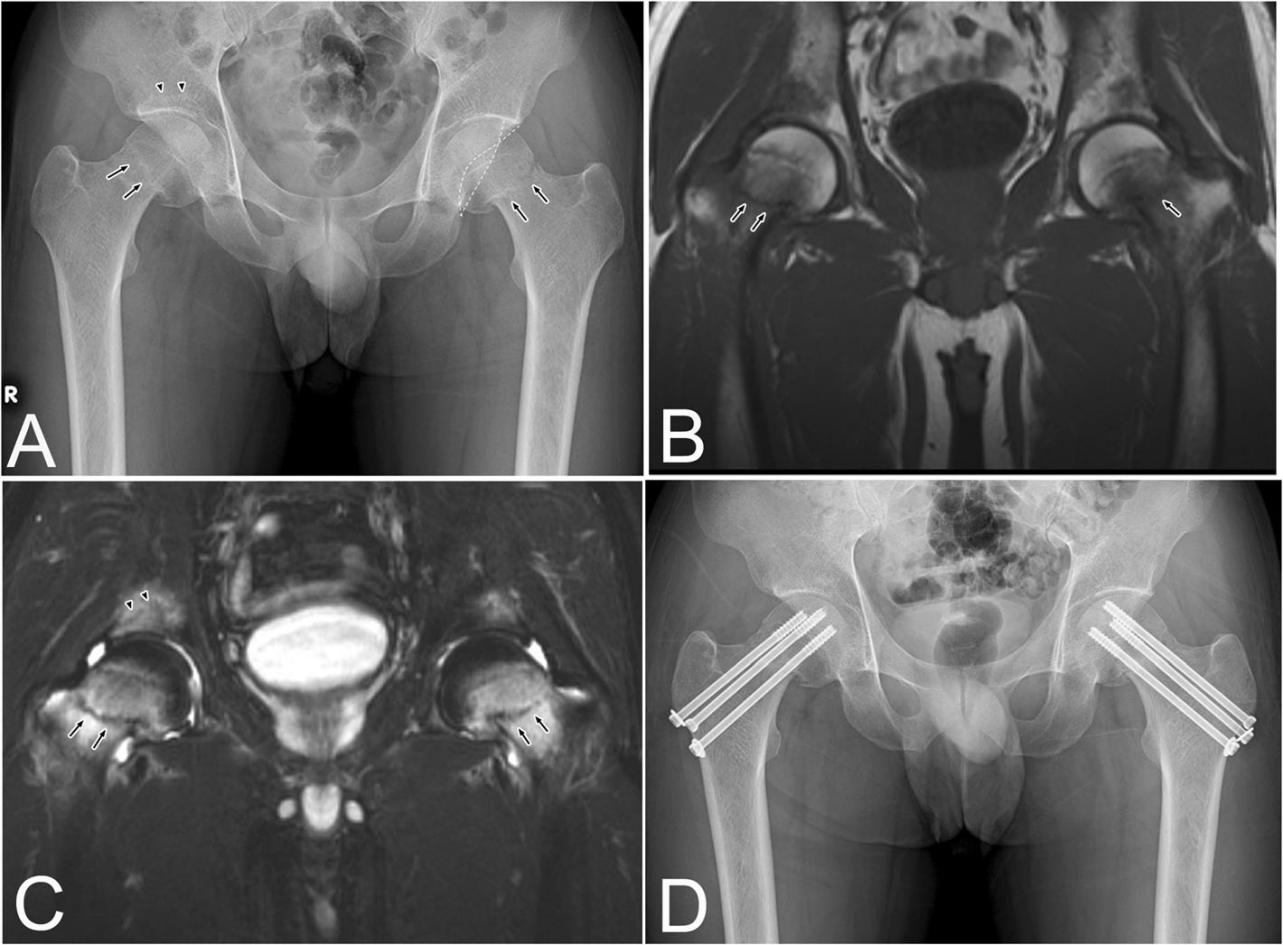
**a** Initial radiograph showing bilateral femoral neck fracture (arrows). The osteosclerotic lesion was observed at the right acetabulum (arrowheads). In the left hip, the cross-over sign was seen (dotted lines). Coronal T1-weighted image (repetition time/echo time [TR/TE] = 474/14 msec) showing diffuse low-signal intensity in the femoral head and lower neck (**b**) and the corresponding high-signal intensity on the T2 fat-saturated image (TR/TE = 3500/87) (**c**). Coronal T1 (**b**) and T2 fat-saturated (**c**) images revealed the fracture line in the bilateral femoral neck (arrows) and in the right subchondral region of the acetabulum (**c**) (arrowheads). **d** Bilateral osteosyntheses using multiple pinning method were simultaneously performed in the bilateral hips

An initial radiograph showed bilateral femoral neck fractures (right: Garden II, non-displaced fracture; left: Garden III, displaced fracture) (Fig. 1a) [12]. Regarding the anatomy, developmental dysplasia was not observed in either hip, but the cross-over sign, which is a radiological sign of acetabular retroversion associated with femoroacetabular impingement, was observed in the left hip (Fig. 1a) [13]. On magnetic resonance images, bone marrow edema was observed at the bilateral femoral neck and acetabulum (Fig. 1b and c). A coronal T2 fat-saturated image revealed the fracture line in the bilateral femoral neck and in the right subchondral region of the acetabulum (Fig. 1c).

**Fig. 2:**
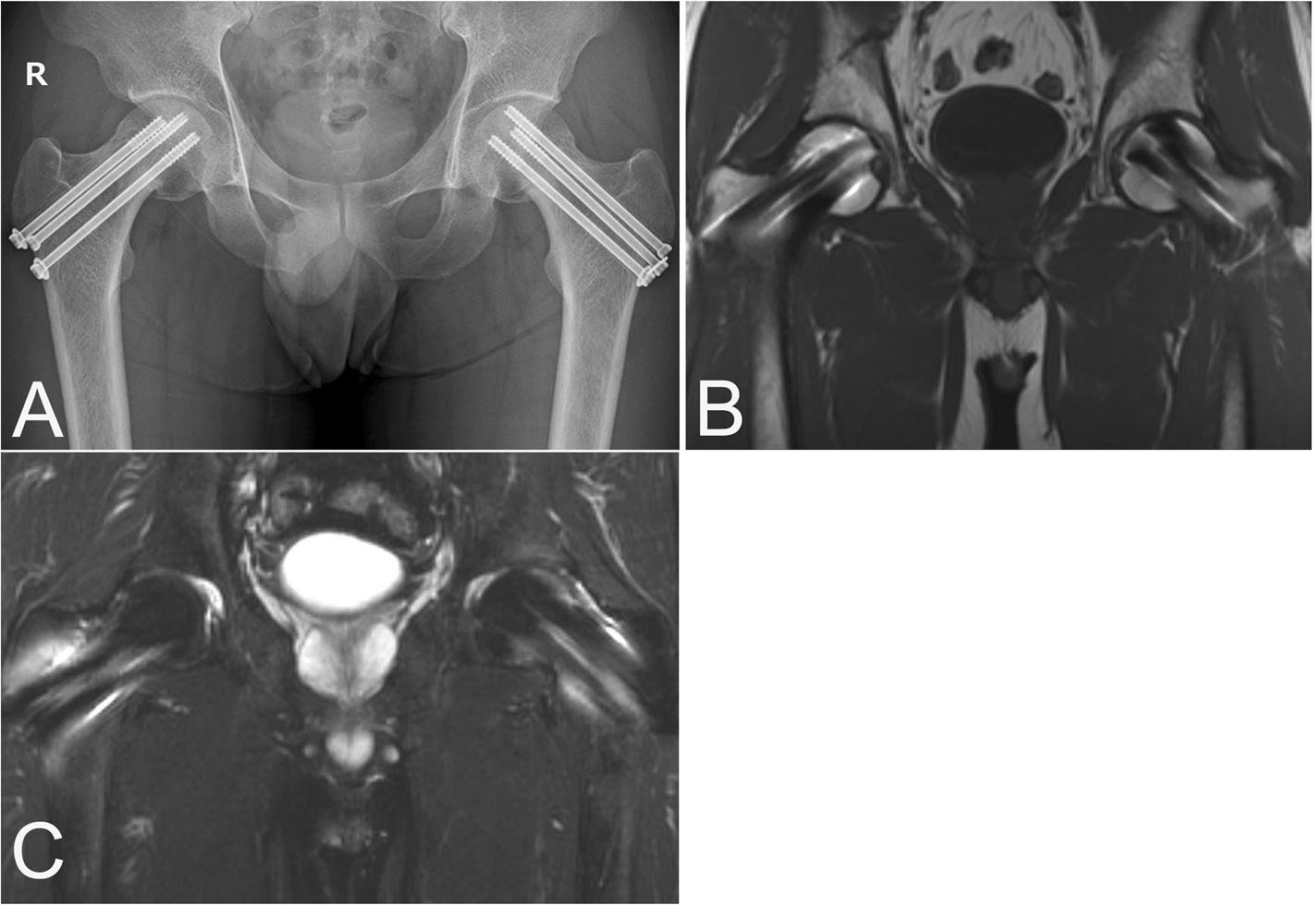
**a** Bone union of the bilateral femoral neck was confirmed by radiography 8 months after the surgery. No abnormal findings were observed on either coronal T1 (TR/TE = 430/14) (**b**) or T2 fat-saturated (TR/TE = 4000/84) (**c**) magnetic resonance images. The bone marrow edema in the bilateral femur and acetabulum disappeared

The patient was diagnosed with simultaneous non-traumatic bilateral femoral neck fractures. Although it was long past the appropriate timing for osteosynthesis (i.e., within 24 h after the onset) [14], bilateral osteosynthesis using the multiple pinning method was performed simultaneously (Fig. 1d) because the patient was young. Postoperatively, walking with full weight bearing and daily subcutaneous injections of teriparatide acetate (20 μg/day) were started soon after the surgery. Oral alfacalcidol (1.0 μg/day) was administered alongside according to safety information [15]. Bone union of the bilateral femoral neck and non-occurrence of osteonecrosis of the femoral heads were confirmed via radiography and magnetic resonance imaging 8 months post-surgery, and the bone marrow edema at the acetabulum disappeared (Figs. 2a-c). The patient’s symptoms also disappeared.

## Discussion and conclusions

Femoral neck stress fractures among young people usually occurs in military recruits and endurance athletes who are subjected to prolonged, high-stress, repetitive lower extremity loading activities [16, 17]. Furthermore, several studies showed an association between femoroacetabular impingement and femoral neck fracture [18, 19]. Although a radiological sign of femoroacetabular impingement (the cross-over sign) was observed in the left hip of our patient, he had just begun a routine of jogging 2 km/ week a month previously. Therefore, it is unlikely that femoral neck stress fracture occurred on both his hips.

Non-traumatic bilateral femoral neck fractures are very rare. Some recent articles reported bilateral femoral neck fractures occurring secondary to transient osteoporosis of the hip in postpartum patients [5, 6]. Although pregnancy-associated osteoporosis occurring during late pregnancy and in the early postpartum period has been reported [20], our case is different from these cases because it involves a young man.

Our case showed severe osteoporosis (T score: − 3.4 SD and − 2.8 SD in the lumbar spine and in the femoral neck, respectively) without apparent underlying metabolic disorders except vitamin D deficiency. Although vitamin D deficiency is prevalent worldwide [21], we consider that the vitamin D deficiency in our case might be partly caused by non-exposure to sunlight for 3 years. Wang et al. [22] indicated a significant positive correlation between serum 25-hydroxyvitamin D and sunlight exposure, but serum 25-hydroxyvitamin D was not correlated with daily vitamin D intake. Therefore, it was thought that the patient’s vitamin D insufficiency may have been caused by his lifestyle. Priemel et al. performed a histomorphometric analysis of iliac crest bone biopsies from 675 patients and reported that bone mineralization defects and pathologic accumulation of osteoid were found when serum 25-hydroxyvitamin D levels were below 30 ng/mL [23]. We believe that vitamin D insufficiency is one of the risk factors for osteoporosis and may lead to non-traumatic femoral neck fracture in young patients who have no underlying risk factors for bone fragility. However, further investigations might be necessary to clarify the comorbidity of vitamin D insufficiency in non-traumatic femoral neck fracture.

Regarding the results of the laboratory tests, the elevated P1NP level might have reflected the effect of the fracture. We administered alfacalcidol in combination with teriparatide for the treatment of severe osteoporosis, partly because alfacalcidol is covered by the insurance in Japan, but not native vitamin D supplements. However, cholecalciferol might be better than alfacalcidol, because patients treated with a combination of teriparatide and alfacalcidol are at risk of hypercalcemia [24].

In summary, we observed that simultaneous non-traumatic bilateral femoral neck fractures can occur in healthy young men with no history of trauma.

## Data Availability

All data concerning the case are presented in the manuscript.

## Abbreviations

CT: computed tomography
MRI: magnetic resonance imaging
SD: standard deviation
